# Trust makers, breakers and brokers: building trust in the Australian food system

**DOI:** 10.1186/1471-2458-13-229

**Published:** 2013-03-15

**Authors:** Annabelle Wilson, John Coveney, Julie Henderson, Samantha Meyer, Michael Calnan, Martin Caraher, Trevor Webb, Anthony Elliott, Paul Ward

**Affiliations:** 1 Discipline of Public Health, Flinders University of South Australia, GPO Box 2100, Adelaide, SA 5001, Australia; 2 School of Nursing and Midwifery, Flinders University of South Australia, GPO Box 2100, Adelaide, SA 5001, Australia; 3 School of Social Policy, Sociology and Social Research, University of Kent, Cornwallis North East, Canterbury, CT2 7NF, KentUK; 4 Centre for Food Policy, Department of Sociology, School of Arts and Social Sciences, City University, Northampton Square, London, EC1V OHB, UK; 5 Behaviour & Regulatory Analysis Section, Food Standards Australia and New Zealand, PO Box 7186, Canberra BC ACT, 2610, Australia; 6 Hawke Research Institute, University of South Australia, GPO Box 2471, Adelaide, SA 5001, Australia

**Keywords:** Food, Trust, Food scare, Food safety, Australia, United Kingdom

## Abstract

**Background:**

The importance of consumer trust in the food supply has previously been identified, and dimensions of consumer trust in food—who they trust and the type of trust that they exhibit—has been explored. However, there is a lack of research about the mechanisms through which consumer trust in the food supply is developed, maintained, broken and repaired. This study seeks to address this gap by exploring if, and how, consumer trust in the food supply is considered by the media, food industry and governments when responding to food scares. The aim of the research is to develop models of trust building that can be implemented following food scares.

**Methods:**

Semi-structured interviews will be undertaken with media, public relations officials and policy makers in Australia, New Zealand and the United Kingdom. Participants will be recruited through purposive sampling and will be asked to discuss a hypothetical case study outlining a food incident, and any experiences of specific food scares. Models of trust development, maintenance and repair will be developed from interview data. Comment on these models will be sought from experts in food-related organizations through a Delphi study, where participants will be asked to consider the usefulness of the models. Participants’ comments will be used to revise the models until consensus is reached on the suitability and usability of the models.

**Discussion:**

This study will contribute to the literature about systems-based trust, and explore trust as a social and regulatory process. The protocol and results will be of interest and use to the food industry, food regulators, consumer advocate groups, media seeking to report food-related issues and policy makers concerned with public health and consumer health and well-being. This research represents an important contribution to the translation of the theoretical conceptualizations of trust into practical use in the context of food.

## Background

Recent research underscores that consumer (dis)trust in food accompanies the experience of food choice [1]; trust and choice are thus intricately interwoven. Trust is a central concept in social science, yet it is one that is complex, contradictory and with manifold connotations [2,3]. Thus whilst the German social theorist Niklas Luhmann argues that trust presupposes knowledge about possible courses of action (which the individual must consciously bear in mind), by contrast the British sociologist Anthony Giddens contends that trust is more of a continuous state – at once psychological and social. Even in this cursory comparison, it is evident that trust is one of those terms in which almost every aspect of its dimensions is problematic.

Trust has been described as habitual, irrational [4], taken for granted, not based on conscious choice [5] but on the assumption that the world will operate as it has before [6]. Trust is, so to speak, thus ‘blind’. Trust is required where there is a lack of knowledge about the trusted by the truster [7], where there is a risk involved in investing trust [8] and where there is also a linked vulnerability on the part of the truster*.* The identification of two kinds of trust in the social science literature, however, appears to be a very general division: of personal and interpersonal attitudes of trust on the one hand, and of dispositions of trust towards institutions and expert systems on the other. Different authors postulate different emphases in analysing this individual/institutional dualism of trust. For example, Giddens [9,10] views emotional and interpersonal trust as essential underpinnings of institutional trust, while Luhmann [11] sees institutional trust relations as a prerequisite to any individual’s trust in a system’s representative. Moreover, in terms of the psychology of trust, it is important to note that social environments can unleash disruptions or breakdowns in trust, either in respect of individuals or complex systems. In this connection, Giddens speaks of the centrality of ‘active trust’ to the age of globalization, and of the ‘emotional regrooving’ that accompanies the experience of disruption to routine social practices [9]. Given the above conceptualization, the notion of trust is of central importance to consumers. Evidence suggests that a proportion of consumers lack *knowledge* about the preparation and procurement of food, food has become increasingly *risky* because of changes in the food industry, and that consumers have become exceedingly dependent on, or *vulnerable* to, the food industry for survival [12-18].

Previous research has explored consumer trust in the food supply, primarily drawing on the extent to which consumers trust the food supply, who they trust and the type of trust that they exhibit (interpersonal or institutional). In general, Australian consumers trust the food supply, with trust for most being habitual because they have no reason to distrust [19]. One study identified that while Australian consumers generally are unable to name the government body that regulates food in Australia and New Zealand, there is a belief that the government is responsible for food regulation [20]. Similarly, young people believed that Australia has satisfactory food regulation, but they could not give specific details about the food regulator [21]. Differing patterns of trust have been found in different types of consumers, with rural consumers being more trusting and more reflexive, demonstrating a greater knowledge base to inform the decision to trust or distrust [17]. It has been argued that the general lack of reflexivity about food choice observed in consumers is a result of a decrease in consumer knowledge about food production caused by recent shifts in food production, procurement and preparation [17]. This was supported by the finding that consumers, except for those living in rural areas, were found to be ‘disembedded’ or disconnected from the food supply [17]. Consequently, the potential for interpersonal trust between a consumer and the food industry is significantly less, suggesting that exploration into consumer trust of food systems is likely to be important.

In terms of who is trusted, Australian consumers have been shown to have a high level of trust in farmers, and moderate levels of trust in supermarkets, politicians and the media when it comes to food [22]. However in terms of truth-telling during a food scare, farmers were the only group that rated moderately well [23]. Other research has shown the media to be an important source of consumer information about food, including food scares [1,16,24], and it plays an important role in shaping the attitudes and consumption habits of consumers [25]. Media reporting has been shown to lead to confusion in some consumers about food safety issues and diet [26] and about food in general [24]. It has been noted that some food-related issues receive widespread publicity in the media while others do not [27], and media coverage of risk is selective [28]. Therefore it is relevant to identify how media construct risk in relation to food.

The role of the government, food industry and media as sources of information about food safety and regulation is evident across the literature. While previous research into food and trust has looked at whether systems-based trust exists, its dimensions have not been explored, in particular the mechanisms through which consumer trust in food is developed, maintained, broken and repaired. Food scares and food incidents provide a good opportunity to explore consumer trust in food in the context of systems, because responses to food scares involve interaction between different systems (for example, media, food regulators) and consumers; potentially creating, maintaining or undermining consumer trust in the food regulatory system [29]. A food incident has been defined as ‘any situation within the food supply chain where there is a risk or potential risk of illness or confirmed illness or injury associated with the consumption of a food or foods’ [30]. The source of the hazard may be microbiological, chemical, radiological, physical or unknown and the incident may or may not attract media or political interest [30]. Furthermore, evidence suggests that exploring food trust in the context of food scares is important; consumer risk perception was shown to affect intention to purchase food during a food scare [29]. Consumer responses to food scares have been shown to be country-specific [29] as has consumer trust in food [31,32]. Therefore it is pertinent to explore the mechanisms of trust making, maintaining and breaking across countries that have experienced different numbers and types of food scares.

The purpose of this study is to explore systems-based trust in the context of food scares across the media, food industry and government, and to examine the role of these organizations in building, breaking and maintaining consumer trust. The study will describe mechanisms through which these systems develop, maintain and break trust with consumers. The findings will be used to develop models for trust development, maintenance and repair that can be used by food-related organizations to facilitate trust with consumers, in the context of food. The purpose of this paper is to report on how this study will be undertaken.

## Methods

### Ethics

Ethics approval was received from the Flinders University Social and Behavioural Research Ethics Committee. This study adheres to the RATS guidelines on qualitative research.

### Setting

This research will be conducted across three countries: Australia, New Zealand and the United Kingdom (UK). Approximately 40 interviews will take place in Australia/ New Zealand and another 40 in the UK. It is pertinent to compare these countries because they have a very different history in relation to food scares, with the UK experiencing a major food scare concerning Bovine Spongiform Encephalopathy (BSE), first identified in cattle in 1986 [25]. Food Standards Australia and New Zealand (FSANZ) is responsible for setting food standards in both Australia and New Zealand; however, in Australia state governments are responsible for enforcement [20], providing a point of difference from the UK for comparison.

### Aims

The aims of this research are to:

• explore why and how food-related organizations (including policy makers, food industry and supermarkets) develop, maintain and rebuild consumer trust in response to food scares,

• explore the role of the media, consumer organizations and public relations departments in developing and maintaining trust during food scares,

• explore and understand trust in food systems in an era of intensive globalization, and

• develop models of trust building, trust maintenance and trust repair.

A sub-aim of this study is to compare the responses from interviews in Australia and New Zealand with those in the UK.

### Study design

The research team consists of members from Australia, New Zealand and the UK who have a wide range of backgrounds including universities, state government and food regulation bodies, indicating the presence of a variety of connections and perspectives. Team members have experience in public health nutrition, food policy, food regulation and sociology. The study design has been informed by our previous research on food and trust (for example [1,17,19,20]) and trust theory [9,11].

The study will occur in three phases, enabling the outcomes from each phase to build upon the next. The purpose of each phase is outlined in Table 1. In Phases 1 and 2 key stakeholders will be interviewed and the information used to develop models of trust building, maintenance and repair. Phase 3 of the research involves a Delphi study. The purpose of a Delphi study is to seek consensus from ‘experts’ about a particular issue, using a series of structured questions (referred to as ‘rounds’) [33]. The responses from participants obtained in each round are summarized and communicated back to participants [33]. In this study, email will be used to conduct the Delphi study. This has previously been shown to be a valuable and rapid method for obtaining expert consensus [34].

**Table 1:** The three phases of the research and the purpose of each phase

**Phase**	**Purpose**
1	Interviews with individuals from the media and public relations organizations within the food industry will identify how they relate and respond to public concerns about food
2	Interviews with policymakers will identify how they develop and set public policy to enhance public trust in food
3	The Delphi method will be used to develop trust-building and trust-repair models to be used by food-related organizations.

### Participants

Key stakeholders will be invited to participate and be interviewed in the different phases. Examples of the types of participant, the organizations or other areas they will be recruited from, and the justification for their inclusion is outlined in Table 2.

**Table 2:** Details of participants

**Phase**	**Actor (n)**	**Examples**	**Sourced from**	**Justification**
1	Media (30: 15 in Australia/ NZ & 15 in UK)	Reporters	Television	Television & newspapers are an important way of conveying information
		Journalists	Newspapers	
		Editors		
	*Trust breakers*	Bloggers (who comment on food/ food regulation)	Internet	Comment on success and failures of food regulation
		Social media/ marketing officers	Food-industry/ food-related organisations	Social media is a way of disseminating food-related information; it can enhance the speed at which communication is sent and received during crises [35] and provides opportunities and challenges for dissemination of information about food safety [36]
	Public relations (PR) officials (30: 15 in Australia/ NZ & 15 in UK)	PR officials working in the food industry/ large food-related organisations	Food industry/ food-related organisations	Could provide ideas of responses specific to food-related organisations & draw on past experiences
		External PR companies previously involved in food scare management	PR companies	Not all food-related organisations will have their own PR staff
	*Trust brokers*	Food safety officers	Food-related organisations e.g. supermarkets, takeaway chains	Could provide an idea of specific responses/strategies used by companies to make/maintain consumer trust
		Organisations that promote consumer interest	Consumer organizations	May have a role in guiding consumer trust in food (promoting trust or distrust)
2	Policy makers (20: 10 in Australia/ NZ and 10 in UK)	Individuals of various seniority levels from national food regulatory bodies	Food Standards Agencies	Set national guidelines for food regulation. Liaise with state-based offices.
	*Trust makers*	State-based food regulators	Health departments	Deal with day-to-day recalls
3	Delphi study (100: 50 in Australia/ NZ & 50 in UK)	Individuals from all groups in Phases 1 and 2 including policy, industry peak bodies, public relations groups and media	CEOs in food-related organisations	Individuals can comment on the suitability of the trust models developed

### Recruitment

Participants will be recruited through purposive and snowball sampling. The research team will primarily use their networks to recruit the participants outlined in Table 2 and key contacts will be asked to suggest other relevant people (snowball sampling). Sampling will be purposive to ensure participants can comment on trust in the context of food scares. Purposive sampling ensures that participants are information rich [37] and is consistent with one of the markers of quality in qualitative research, sampling via relevance [38].

### Data collection

(a) Participant interviews

An interview schedule has been developed for participant interviews in Phases 1 and 2 (Table 3). It was developed to investigate social theories of trust and evident research gaps identified in our review of the literature. An important component of the interview schedule is the hypothetical case study (Table 4), designed to facilitate comparisons between countries. It covers a wide range of issues that may arise during food scares, including widespread effects, vulnerable groups affected and potentially serious health implications. Because of the dynamic nature of food industries and food scares across the three countries, it was not possible to investigate a real case. Previous studies have used a similar approach in order to guarantee consistency in consumer responses across countries [29]. The case study will be presented at the start of the interview as a way of exploring hypothetical responses to food scares with each participant. It will be followed by general questions, including some that participants will be asked to think about in the context of a food scare that they have experienced. These will enable reflection on lessons that can be learned from real life situations. Examples of the questions to be asked in interviews, in particular those related to the case study, are included (Table 3). Interviews were chosen to allow in-depth exploration of participant’s perceptions and practice about trust and food in a one-to-one situation. The identity of interview participants will be kept confidential.

In Australia and New Zealand, interviews will be conducted by the Research Fellow (AW) and in the UK by a research assistant. Each interview will last up to 60 minutes and will be recorded digitally and transcribed verbatim.

(b) Development of trust models and Delphi study

Models of trust development, trust maintenance and trust repair will be developed from information provided in interviews. The Delphi method will be used to arrive at a consensus among experts about the suitability of these models. The Delphi study will be conducted concurrently, but separately, in Australia/ NZ and the UK. It will occur after Phases 1 and 2 and will involve two rounds. In Round 1, the proposed trust models and a questionnaire will be emailed to participants. The questionnaire will ask them about their views on the different trust models, their experiences of using any of them within their organization and their views on the feasibility of using each of the models in their organization in the future. Findings from Round 1 will be used to refine the models. In Round 2 the revised models will be sent to participants, who will be asked to respond to the changes, either by accepting the models as they are or by suggesting further changes.

**Table 3:** **Hypothetical case study to be used in interviews for the study *Trust makers, breakers and brokers: building trust in the Australian food system***

Case study	Elements
	- Large food manufacturer has identified contaminated soy protein isolate during routine testing of raw ingredients
	- Source of contaminated soy protein isolate is an Asian country
	- Soy protein isolate is used extensively in the food industry to increase the protein content of a wide variety of foods and drinks that are consumed across all age and social groups
	- Soy protein isolates are also used in infant formulas
	- Subsequent testing has identified the contaminated soy protein isolate in leading brands of infant formula, breakfast cereal, bread and other products that are currently on sale
	- The contaminated product is potentially hepatotoxic, containing a toxin that causes acute liver disease
	- Literature suggests that the toxin can be fatal in vulnerable groups such as children, pregnant women and older people

**Table 4:** **Interview schedule to be used in the study *Trust makers, breakers and brokers: building trust in the Australian food system***

**Group**	**Example questions**
Media	• What would make this story newsworthy?
	• Would you run with this story? Why or why not?
	• What is the immediate story? What are the underlying issues that the media would follow up?
	• What key words would you put in your headline? What angle would you take on the story?
	• What sources would you seek and why?
	• What would you draw on to frame/ anchor the story?
	• What risks would you identify in this case that you would seek to convey to consumers?
	• What reaction would your story elicit in consumers?
	• What impact do you see your story/ reporting having on consumer trust?
Public relations officials	• Discuss the extent to which this is a realistic scenario
	• Discuss whether this scenario is likely to be significant issue for the company concerned. If so, what features are salient?
	• How would you respond to a situation like this?
	• Are issues of public trust or confidence in the food supply considered in dealing with this issue? What would you do in this situation to facilitate trust with consumers?
	• What responsibility do you think, if any, that media consider when publicising this story?
Policy makers	• To what extent is this a realistic scenario?
	• Is this situation likely to be significant issue for the company concerned? Why or why not? What features are salient?
	• How would you respond to a situation like this?
	• How important is trust in food policy setting and decision making?
	• What specific mechanisms are used by policy makers to enhance consumer trust in the food system?
	• What processes of trust building currently exist between policy makers and consumers? What are the strengths and weaknesses of these current processes?
	• How could policy be used to facilitate building and maintaining consumer trust?
	• Do you use social media to communicate with consumers (for example in order to inform them of policy)? Why or why not? Are you targeting a particular consumer group?
	• What platforms (Facebook, Twitter, blogs, other) do you use? When do you use them?

### Data analysis

Data analysis will be informed by a grounded theory approach [39]. New data will be compared and contrasted with earlier findings to look for common themes and highlight differences in how organizations and groups relate to food scares. NVivo software will be used to organize data. Data from interviews will be used to develop the models of trust building, maintenance and repair.

## Discussion

This research will add to the theoretical conceptualizations of systems-based trust. While existing trust theories offer multiple ways to understand trust, current models do not explore trust as a process; that is, the mechanisms or processes that are required to build, repair and maintain trust. The lack of information about trust as a process is compounded by the multiple definitions of it, which make investigation difficult. For example, the process of making, maintaining and breaking trust will be different when using Luhmann’s definition of trust, which requires agents to have some knowledge and be reflexive [11], compared with the idea that trust is habitual and taken for granted [5] and automatic, assuming that the world continues to operate as it has before [6]. This study will provide insight into the mechanisms by which trust is developed, maintained, broken and rebuilt with consumers in the context of food scares, and develop practical models that outline these processes. It will consider if, and how, different theoretical perspectives of trust can be accounted for in trust models. This study also has the potential to allow the introduction of other theoretical understandings to complement and extend those perspectives provided by social theories of trust, including theories of risk.

Development of models is a way by which social theories of trust can be translated into practice in the food setting, thus extending theory into public health policy and practice. That is, trust theories will assist in contextualizing the processes of trust identified in interviews, which in turn will allow development of the models. Individuals working in the food system, such as food industry, policymakers and public relations departments can address the processes identified in the models in order to build and maintain trust, hence maximizing the trust of consumers in the food supply. This is particularly relevant in the case of future food scares, where these models will enable food regulators, the food industry and public relations officials involved in food scares to respond to food scares in a way that promotes consumer trust. By obtaining a media perspective, the research will provide an indication of how food scares are framed by the media, which will enable development of trust models that take account of this. The ideal outcome during a food scare, through the use of these models, would be that consumers are accurately informed about the food scare and are able to take appropriate action. Ensuring that trust is (re)built is important because trust affects food choice [1]. Our previous research has identified only moderate levels of trust in supermarkets, politicians and the media with regard to food [19]. This may indicate that consumers may be unlikely to respond to food scares appropriately if they distrust the message or messengers. Additionally, if a food incident is related to a major nutrient group (e.g. BSE in beef) then there could be nutritional implications for a population at a public health level. Of further importance is an investigation into how these models may be relevant and used in different settings and with different consumer groups, as it has previously been identified that an individual’s willingness to trust is dependent on social factors such as socio-economic status, class and age [10]. The cross-national comparisons will contribute to this by enabling identification of whether models of trust building, maintenance and repair are similar or different across countries with different circumstances. This is important because of the different histories of the countries with respect to food scares, and because it has been identified that previous experiences with food scares can affect consumer trust [31].

This study design is strengthened by the collective involvement of researchers, policy makers, theoreticians and food regulators from Australia, New Zealand and the UK. This provides multiple perspectives and ensures that the voices of representatives of those who may use the models can be heard. An area for future exploration is whether the models are well received by consumers, and how they could be incorporated into policy, for example the *National Food Incident Response Protocol*[30]. The advantages of the Delphi technique include consultation with experts who are separated geographically, giving participants time to consider responses, the avoidance of one or two persons dominating within the group as can happen in focus groups, and providing people with opportunities to comment individually while still using a group to discuss ideas [40,41]. However, it is important to keep in mind that the results from a Delphi study identify consensus on an issue using that particular group of experts. In this study, we will include experts from three different countries (Australia, New Zealand and the UK) and from different areas (policy, industry peak bodies, media, public relations staff and food-related organizations) to ensure that a wide variety of perspectives can be heard. Particular attention also needs to be paid into structuring group discussion and raising points for discussion to ensure that a wide range of issues are considered and discussed [33].

In conclusion, the protocol and results of this research will be of interest and use to the food industry, food regulators, consumer advocate groups, media seeking to report food-related issues and policy makers concerned with public health and consumer health and wellbeing. The findings will facilitate connections between research, theory and policy and will help to fill a gap in the translation of trust theory into public health practice, in the context of consumer trust in food.

## Competing interest

The authors declare that they have no competing interests.

## Authors’ contributions

JC, PW, JH, SM, TW, AE, MC and MC contributed to the conceptualization of the project and to the initial grant application. AW wrote the first draft of the manuscript and it was reviewed by all authors. All authors read and approved the final manuscript.

## Pre-publication history

The pre-publication history for this paper can be accessed here:

http://www.biomedcentral.com/1471-2458/13/229/prepub
